# Smart Contract Broker: Improving Smart Contract Reusability in a Blockchain Environment

**DOI:** 10.3390/s23136149

**Published:** 2023-07-04

**Authors:** Joonseok Park, Sumin Jeong, Keunhyuk Yeom

**Affiliations:** 1 Research Institute of Intelligent Logistics Big Data, Pusan National University, Busan 46241, Republic of Korea; pjs50@pusan.ac.kr; 2 Department of Information Convergence Engineering, Pusan National University, Busan 46241, Republic of Korea; sumin2708@gmail.com; 3 School of Computer Science and Engineering, Pusan National University, Busan 46241, Republic of Korea

**Keywords:** smart contract, smart contract broker, smart contract management, blockchain

## Abstract

In this paper, we propose a smart contract broker to improve the reusability of smart contracts in a blockchain environment. The current blockchain platform lacks a standard approach to sharing and managing smart contracts, which makes it difficult for developers to reuse them and leads to efficiency issues. The proposed smart contract broker uses tags to identify and organize smart contracts, and it provides an environment for comparing and reusing smart contracts. This improves the reusability of smart contracts and efficiency. The proposed smart contract broker can be applied as a reference model that increases the flexibility and reusability of smart contract management in a blockchain environment.

## 1. Introduction

In this paper, a smart contract broker is introduced to improve the reusability of a smart contract in a blockchain [1] environment. Smart contracts are programs written in languages such as Go [2] and Python [3], and stored on a blockchain for automating the execution of an agreement. They are applied as a key technology [4] to realize the immutability, transparency, and efficiency of blockchains in various domains such as finance, smart cities, and voting. When a business transaction occurs on the blockchain, the smart contract is executed automatically. For example, smart contracts can be used to trace the movement of goods and materials through a supply chain. 

However, there are issues, such as the difficulty of use, lack of convenience, and lack of a support environment when applying smart contracts to Hyperledger Fabric, a leading permissioned blockchain platform. The complexity and usability concerns of open-source permissioned blockchains, such as Hyperledger Fabric [5], have inhibited the configuration of the core blockchain network. Moreover, prior understanding of the Fabric network’s command line interface (CLI) commands, flags, and other necessary vocabulary is required. In addition, the network lacks a method for sharing smart contracts [6,7,8] for transactions between blockchain peers (users) [9] or allowing other blockchain peers to reuse smart contracts. 

Therefore, in this study, an approach named smart contract broker is introduced to improve the reusability of a smart contract in a blockchain environment. The proposed smart contract broker consists of a smart contract management (SCM) system that sup-ports the sharing of smart contracts, a dashboard that can be used as an interface between the users and shared smart contracts, and a broker system that enables connection to the blockchain network. The proposed methodology stores and manages the user, structure, and asset information of the smart contract, as tags, for management. 

The proposed smart contract broker system will make it easier to share smart con-tracts between users. This will improve the reusability of smart contracts and make it easier to develop new applications. The dashboard will provide a user-friendly interface for interacting with smart contracts. The broker system will ensure that smart contracts are connected to the blockchain network and can be executed securely.

The smart contract broker is a promising approach to improving the reusability of smart contracts in a blockchain environment. The broker has the potential to make it easier to develop new smart contracts by comparing and reusing part of existing smart contract. 

The paper is structured as follows: Section 2 reviews related work, and Section 3 introduces the architecture of the proposed smart contract broker and suggests a method for managing smart contracts. Section 4 presents case studies and evaluations of the smart contract broker, and Section 5 discusses practical implications. Section 6 provides the conclusion and briefly describes future research directions.

## 2. Related Work

### 2.1. Permissioned Blockchain—Hyperledger Fabric

Hyperledger Fabric [10], the representative permissioned blockchain platform, has proven to be beneficial for many enterprises because of its wide feature set and active development community. It is a popular open-source permissioned blockchain platform with modularity and a versatility-focused design. The latest version of Fabric as of March 2023 is 2.5.0-beta2. Hyperledger Composer [11]—also part of the Hyperledger Foundation—is an open-source development toolset for simplifying application development on Fabric. Although Composer provides REST (representational state transfer) server functionality, it was officially deprecated on 29 August 2019, and only supports Fabric version 1.4 and earlier. According to the deprecation of Composer, the usability of the blockchain network and interface issues have been ignored, and essential API services are missing.

### 2.2. Smart Contract

In this paper, issues related to representative permissioned blockchains, as discussed above, are presented, with a focus on researching smart contract management methods that can interoperate with them.

Smart contracts have various functions that correspond to the contents of the contract. Smart contract functions can be developed in a programming language that supports Java Virtual Machine (JVM) or Node.js [12] runtime. Moreover, various functions and control statements exist such as if…else or while, that process transaction data and status in a blockchain environment. In Hyperledger Fabric, a smart contract is defined within a chaincode. The smart contract is a key element in the blockchain network, and various studies [13,14] such as smart contract design [15] and application development [16] have been presented. Zou et al. [17] identified the lack of a plan for supporting the evolution, management, and deployment of smart contracts as one of the open research topics to be addressed. In Table 1 of Section 2.3, Discussion, we have presented a summary of the related papers that deal with various issues concerning smart contracts.

### 2.3. Discussion

We have focused on solving the difficulties of managing smart contracts related to the smart contract broker proposed herein. Wu et al. [14] proposed a smart contract life cycle comprising contract generation, contract release, and contract execution. They proposed the overall life cycle from a programmatic perspective, including steps such as creating a contract specification through the negotiation of contract participants and preparing a code contract, as shown in the contract generation stage. This paper also proposes a mechanism that allows smart contracts to be registered and searched for, based on tags, so that a new smart contract can be generated by reusing an existing smart contract. Further, it proposes a mechanism to automate the execution of smart contracts by deriving an API that can provide smart contracts in linkage with blockchain.

In Table 1, we have summarized papers that have analyzed the emerging issues in current smart contracts from the perspectives of the main objective, issues, and future trends.

As is shown in Table 1, smart contracts have been studied for various research purposes, including optimization, modeling methods in blockchains, resources for contract composition, and formalization methods. The issues of smart contract technology include resource immutability, system scalability, formal testing, domain-specific language (DSL), smart contract management, standardization, and verification. The analysis shows that these technical issues arise because smart contracts do not have a standard model or a unified language form, and have separate resource models and specialties for each platform.

Owing to a lack of smart contract management measures, this study aimed to contribute to the creation of a standard method of generating smart contracts and smart contracts that are generated as needed in the business domain. To this end, we propose a smart contract broker to increase reusability when generating smart contracts. Furthermore, we suggest a basic environment that can support the distribution of smart contracts in the blockchain environment by applying the RESTful API [21,22].

## 3. Smart Contract Broker

### 3.1. Smart Contract Broker Architecture

**Figure 1:**
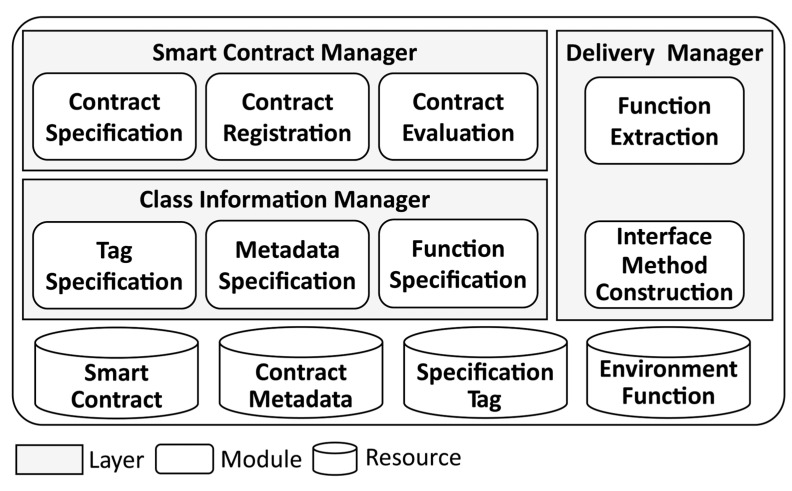
Management Functionality Architecture.

Figure 1 shows the definition of the functionality architecture for performing smart contract management.

The functional elements of smart contract management in Figure 1 were defined to perform the role of the smart contract broker proposed herein by re-specifying smart contracts and metadata that constitute smart contracts. This was accomplished using a series of processes: creating, distributing, and operating smart contracts, and adding steps to evaluate smart contracts. Each type of manager and resource are described as follows:

Smart contract manager: This performs the role of defining smart contracts and preparing for their distribution on the blockchain platform. It uses contract specification for smart contract definition, contract evaluation to judge the defined smart contract, and contract registration to reflect the smart contract on the blockchain platform.

Class information manager: This prepares the application for the smart contract management method proposed herein. To classify smart contracts, it creates tags for the management of smart contracts and maps them to smart contracts in the tag specification. It performs metadata specification to specify the metadata, which will be used to manage the smart contract so that external management can be performed. Through function specification, it specifies the functionality information so that it can be checked and managed outside of the smart contract.

Delivery manager: This performs the role of linking smart contracts with the outside world. It performs function extraction to extract each function that manages a smart contract provided by an external blockchain platform and performs interface method construction to establish a connection with the extracted function.

**Figure 2:**
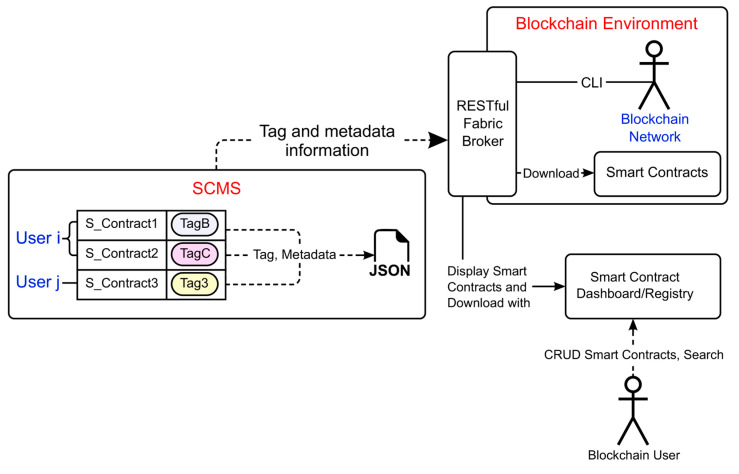
Smart Contract Broker flow architecture.

Figure 2 shows the flow architecture of the smart contract management system (SCMS), which is linked to the blockchain environment presented in this paper. The SCMS performs the role of smart contract manager and class information manager in the functionality architecture of Figure 1, and the RESTful fabric broker performs the role of delivery manager.

SCMs—This is a support system for independently searching and managing smart contracts used in the blockchain network. As is illustrated in Figure 2, tags can be used to group the smart contracts into various types, such as by user and programming language. Furthermore, using JSON (JavaScript object notation) [23], the smart contract is designed to enable internal and external information transfer.

Smart contract dashboard—This is the dashboard in which the user can access the SCM system and blockchain network. Using this dashboard, smart contracts can be searched, downloaded, compared, uploaded, and automatically installed onto the blockchain network based on tags. 

RESTful Fabric broker—This connects blockchain network and system components through REST API and processes the requests. Blockchain functions, which is a component of the broker, supports commands executed in the blockchain network to be executed through API. Furthermore, external functions increase system scalability by linking to other systems, e.g., the dashboard and tagging user input information.

Blockchain network—This refers to blockchain platforms, such as Hyperledger Fabric and Ethereum, in which smart contracts are processed. 

**Figure 3:**
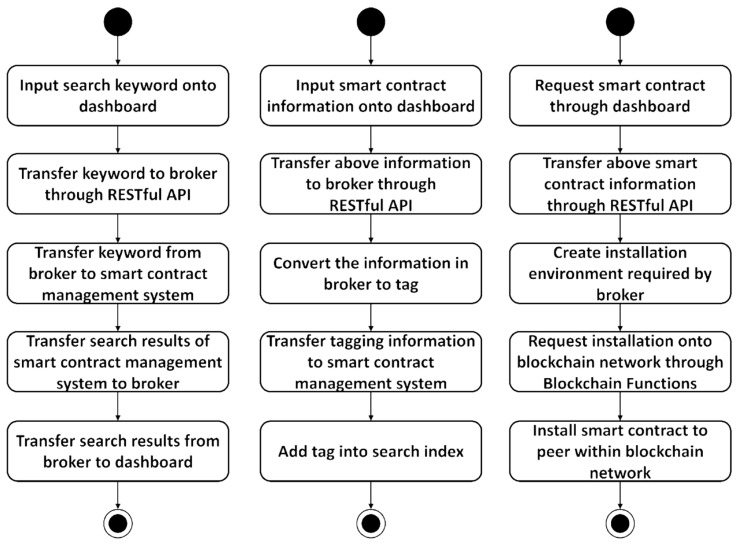
Flowchart of smart contract search (**left**), upload (**center**), and installation on the blockchain network (**right**).

Figure 3 shows the process of searching for, uploading, and installing smart contracts onto the blockchain network.

### 3.2. Smart Contract Management 

**Figure 4:**
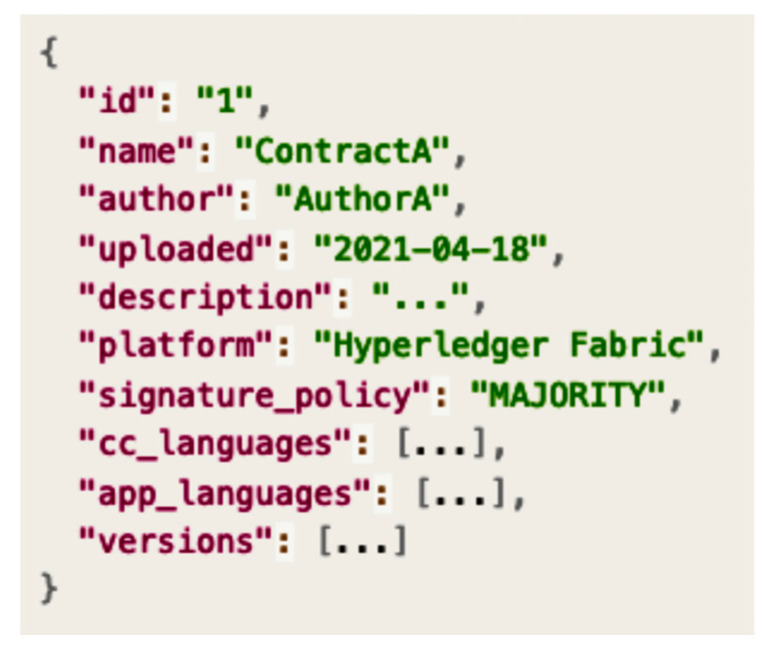
Sample expression of smart contract tag information.

A tag is used to express the information of the smart contract, such as metadata and user information, in the form of key value. The tag expression factors include the name of the smart contract, creator, and blockchain transaction. Figure 4 presents an example of tag expression. 

**Figure 5:**
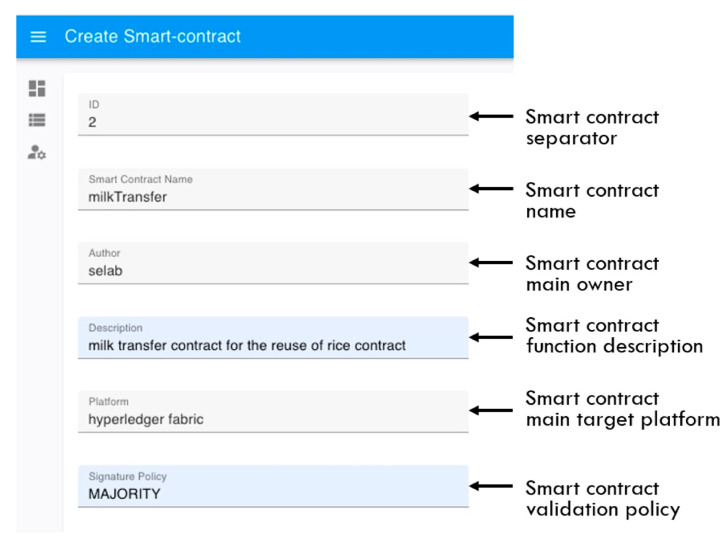
Upload specification for smart contract metadata.

**Figure 6:**
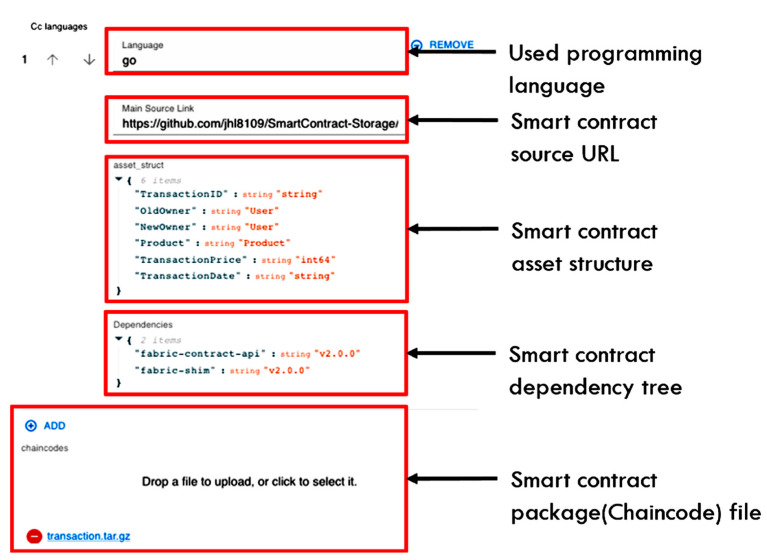
Upload specification for smart contract implementation data.

**Figure 7:**
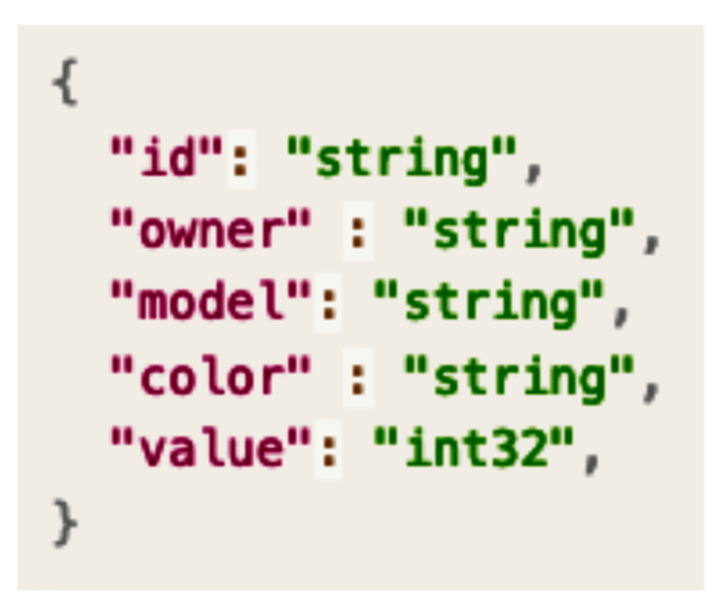
Asset structure of Contract A.

Figure 5 and Figure 6 show the information specified for smart contract upload to the SCM system according to the concept of the proposed tag. The smart contract upload specification information proposed in this paper is divided into a metadata specification tag and an implementation specification tag. Figure 7 shows a metadata specification tag for smart contract management, including the smart contract identifier (ID), name, owner, description, target platform, and contract basic authentication specification. The metadata specification tag describes the classification of smart contracts and basic information for smart contracts to be executed on the platform.

When managing smart contracts, the tag information is managed and the cost of smart contract functions is computed according to the criteria (see Table 2) [24], in order to improve the execution and stability management of smart contract transaction processing.

Table 2 details the factors defined in this paper for determining the execution cost of smart contract functions. The execution cost of each smart contract function is defined and prioritized according to various factors affecting the execution time of the function. 

The code length is assigned a unit cost for every *N* line. As the lengths of the functions in the smart contract vary, *N* is arbitrarily set so that the functions can be distinguished. Control statements are included in the calculation of the execution cost because it directly affects function execution. Data processing refers to the functions that change data according to a desired format or read and write values on a ledger. Data processing functions are included in the calculation of the execution cost because it can also affect the execution time.

Execution cost is the cost allocated to each factor, in which the cost of all factors is set to 1 and the cost is added whenever the factor appears. The lower the sum of all costs, the shorter the execution time. 

Weight is the weight to which each element is compared. If the final cost is the same, the operation with the largest weight is compared.

After the smart contract is registered, it searches for similar smart contracts to be reused by performing a search based on the tag. For example, a seller confirms a relevant asset structure in the searched smart contract. Figure 7 shows a sample output of an asset structure. 

**Figure 8:**
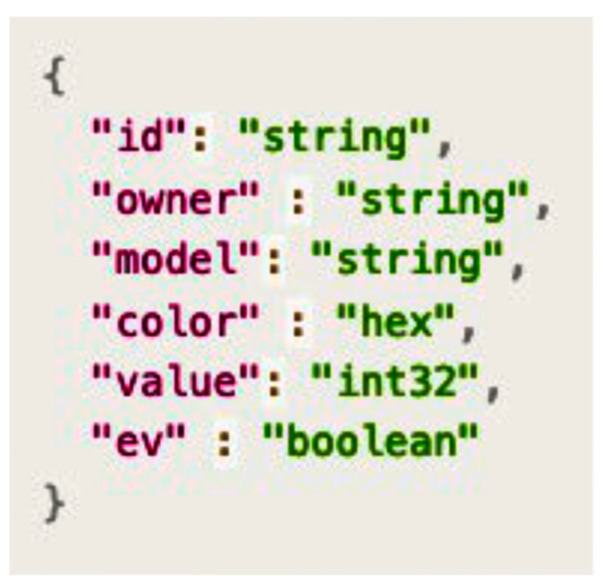
Asset structure of Contract B.

The seller may want to change the asset structure, in which color is to be expressed in hex (hexadecimal) and a Boolean field called electric vehicle (EV) is to be added. Figure 8 shows the asset structure requested by the seller.

The user downloads the smart contract (Contract A) and subsequently modifies the asset structure and creates a new smart contract (Contract B) with the code inserted based on requirement.

**Figure 9:**
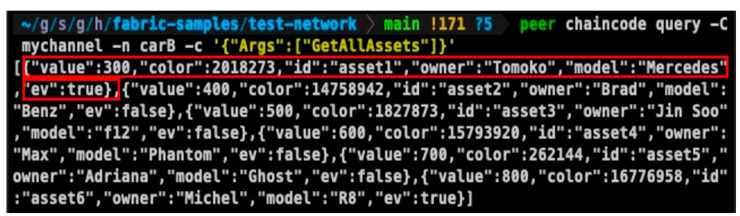
Actual blockchain assets using the asset structure of Contract B.

**Figure 10:**
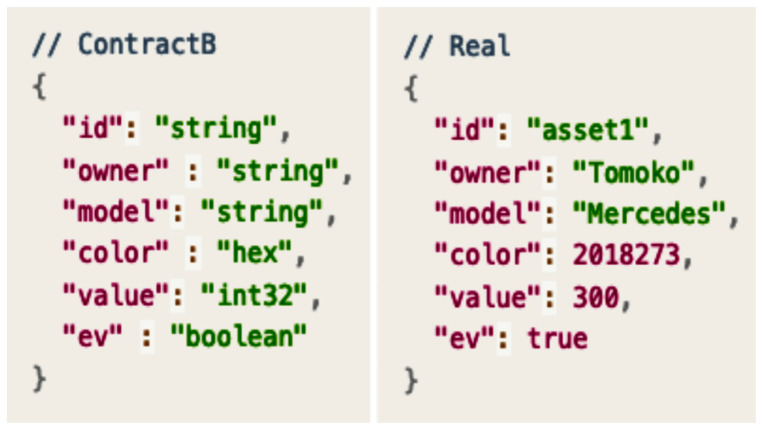
Comparison between the asset structure of Contract B and an actual asset.

After the smart contract installation is complete, the blockchain network can be used to investigate whether the smart contract executes properly, as shown in Figure 9. In Figure 9, the asset1 vehicle indicated in the red box is an electric Mercedes model and the owner is Tomoko. Figure 10 shows a comparison between the vehicle information and the asset structure of Contract B.

### 3.3. API for Smart Contract Management

The broker contains five modules that focus on specific functionality. Each module has a RESTful API that fits the functionality of the module. Table 3 shows the name and description of each module, the method of the implemented API, and the features of each method.

**Figure 11:**
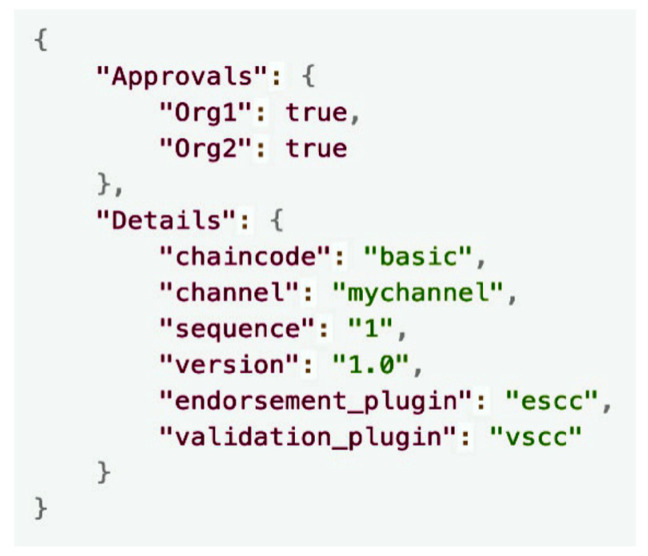
Result of the queryCommittedCC request.

Figure 11 shows the result of executing the GET/fabric/lifecycle/commit from Table 3. This response from the broker provides details regarding the committed chaincode, including its name and the channel to which it was committed.

## 4. Case Study and Evaluation

### 4.1. Case Study

An example of applying a supply chain management [25] smart contract using a smart contract broker was considered. The presented case study describes the process and system of reusing a supply chain management smart contract that trades rice and milk based on the implemented smart contract broker prototype. The progress of the case study consists of uploading the smart contract, searching the smart contract, verifying reusability via a comparison of search result assets, deploying the smart contract on the blockchain network, and executing the transaction result.

**Figure 12:**
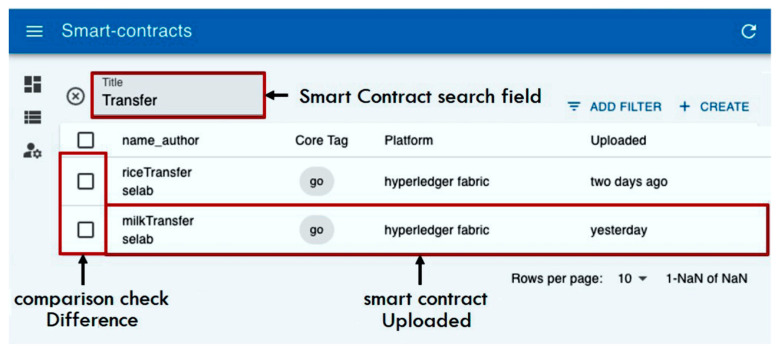
Search results displayed on the dashboard.

An example of retrieving a smart contract provided by a smart contract broker based on the upload specification is shown in Figure 12, which shows the dashboard result screen searched with the keyword “Transfer”. The search result can check the smart contract name, author, and tags such as the writing program language, contract platform, and upload time.

**Figure 13:**
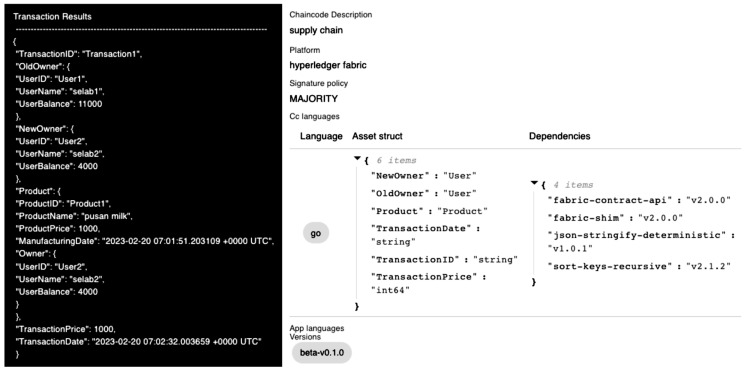
Uploaded detailed view; transaction results (**Left**) and smart contract specification (**Right**).

Figure 13 shows that the smart contract is deployed and executed on the blockchain network after uploading and searching for the smart contract. Smart contract specification appears in dashboard (right) and the transaction result appears as a box by executing the smart contract (left).

**Figure 14:**
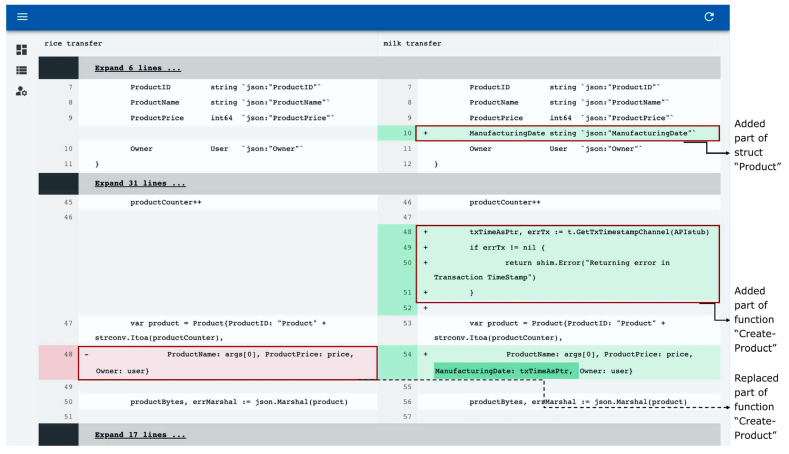
Smart contract difference comparison check.

Figure 14 shows the derivation of “milkTransfer,” a milk transaction management smart contract, based on “riceTransfer,” a rice transaction management smart contract that already exists on the platform. As is shown in Figure 14, the code for reusing assets used in rice transactions and additionally processing reused assets has been extended in the smart contract.

**Figure 15:**
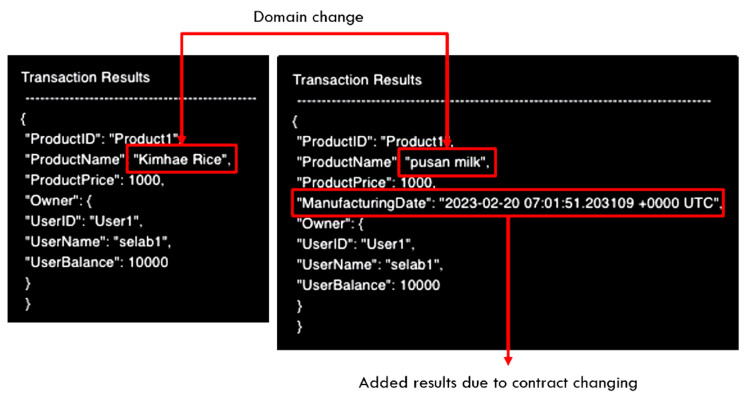
Transaction result comparison (**left**: riceTransfer, **right**: milkTransfer).

In addition, the comparison of smart contract execution results by smart contract transaction domain is shown in Figure 15. Transactions are performed based on the basic information in the existing rice transaction management smart contract. In the case of milk transactions, additional information exists (e.g., freshness); thus, the production date is added as shown on the right of Figure 15 in the transaction result.

### 4.2. Evaluation

**Figure 16:**
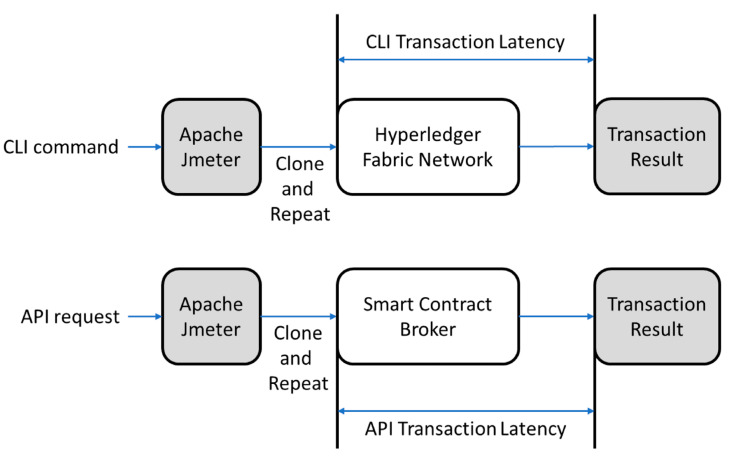
Transaction evaluation.

The performance evaluation of the smart contract broker (see Figure 16) is performed by CLI on the existing permissioned platform and API request of the smart contract broker. After replicating and repeating the unit test with Apache Jmeter [26], an open-source unit test, the latency of each transaction is measured. The performance evaluation was conducted using the smart contract written in the case study.

**Figure 17:**
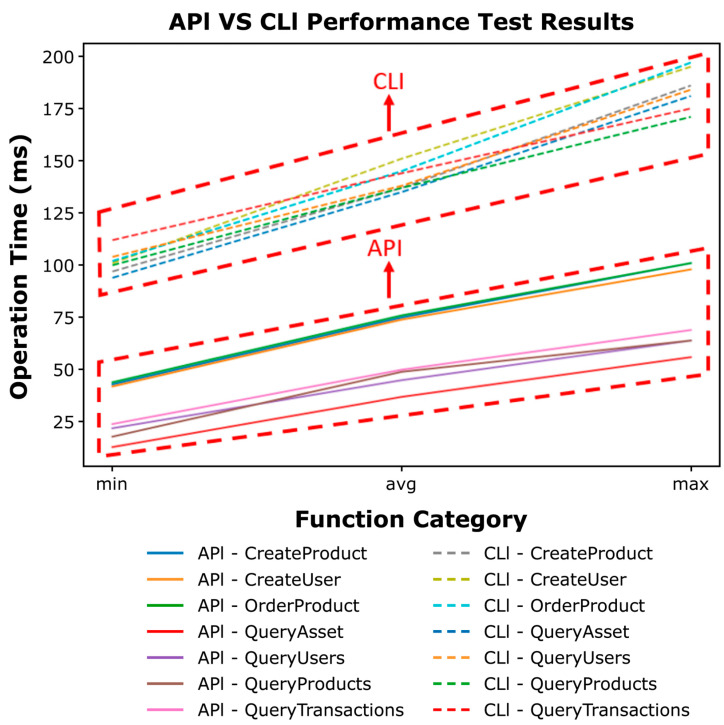
API, CLI performance test results.

**Figure 18:**
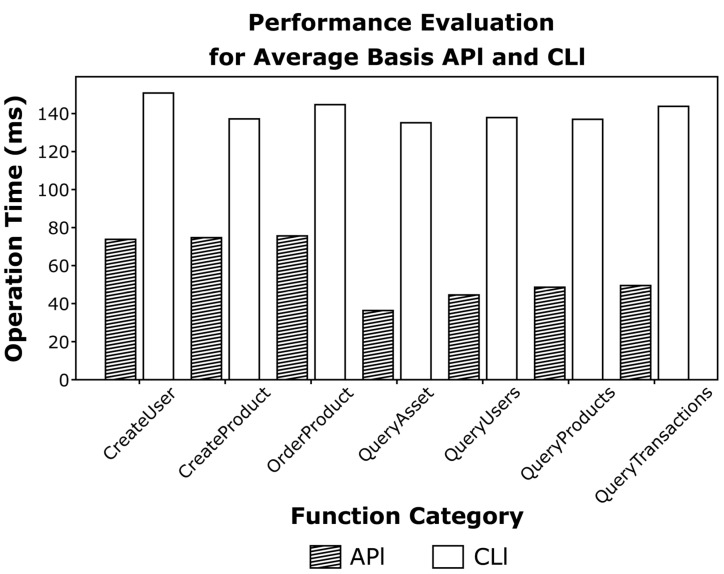
Performance evaluation based on average latency.

The performance evaluation results are shown in Figure 17 and Figure 18. Figure 17 presents a comparison of the minimum, average, and maximum delay times of each method when 100 transaction requests are performed for each smart contract method using CLI and API.

Figure 18 shows a comparison of the average execution time of the CLI and API methods. API execution applied to the smart contract broker improved performance by 60 to 90 ms compared to CLI execution.

## 5. Practical Implication

The key features of the proposed smart contract broker are described below:

- Tag-based management: Smart contracts can be registered, searched, deployed, and executed using tags. This makes it easy to find and reuse smart contracts that are relevant to a particular application.
- Asset structure comparison: The smart contract broker can compare the asset structures of similar smart contracts. This can be used to identify similarities and differences between smart contracts and to reuse parts of existing smart contracts to create new smart contracts.
- REST API: The smart contract broker can be accessed using a REST API. This makes it easy to integrate the smart contract broker with other applications and services.

First, based on the presented features, developers can use the smart contract broker to create smart contracts that perform business transactions specific to the application domain. This may produce the effect of reducing costs by reducing the smart contract creation time. Second, the smart contract broker can be used as a smart contract search engine or smart contract market. As tag information is specified and managed in the created smart contracts, it can serve as a search engine for developers to find usable smart contracts and can be applied as a market for trading smart contracts in the future. Third, it can be used as a blockchain test bed. It provides an environment where users and companies intending to adopt and test blockchain can execute and test smart contracts using APIs and dashboards without specialized knowledge. 

## 6. Conclusions

In this paper, a smart contract broker was proposed to provide a mechanism to increase the reusability of smart contracts and support their management. The concept, structure, and application technique of a smart contract broker to support the management and reuse of smart contracts in a blockchain environment were presented. In addition, a prototype was developed by applying this concept. Case studies show that smart contract management and reuse based on smart contract brokers are applied. Further, rather than executing a smart contract via the CLI provided in the blockchain environment, the execution performance improved using the API of the proposed smart contract broker. 

The contributions of this paper are as follows: (1) We demonstrated that the efficiency of smart contract search and management can be improved by classifying smart contracts based on tags; (2) We proposed a mechanism for reusing existing smart contracts to reduce the cost of creating new smart contracts; (3) We showed that the usability and accessibility of smart contracts can be increased by applying REST APIs. Therefore, the smart contract broker may facilitate access to blockchain networks while providing developers with a systematic and flexible method of managing and reusing smart contract in the blockchain network. This can increase the use of blockchain networks via transaction efficiency between peers and user convenience. In the future, we plan to study a mechanism for collecting and managing the access history of users who search and register based on tags in the current smart contract broker. Based on this, we plan to propose a smart contract broker technology to recommend smart contracts that meet the user’s purpose.

## Figures and Tables

**Table 1:** Literature review of papers on smart contract.

Study	Main Objective	Issues	Future Trends
Khan et al. [18]	Smart contract optimization, blockchain environment modeling, smart contract resources etc.	Resource immutability, system scalability etc.	Layer2 protocol (network issue), contract management
Singh et al. [19]	Smart contract formalization, smart contract vulnerabilities	Formal testing, domain-specific languages	Formal verification of smart contract
Wang et al. [16]	Blockchain architecture, smart contract relationship	Smart contract management etc.	Formal verification of smart contract
Ante et al. [20]	Classification of technical elements of blockchain smart contract system	Smart contract standardization, verification etc.	Layer2 protocol (network issue), definition of smart contract, infrastructure

**Table 2:** Weight and criteria for determining the smart contract function execution cost.

Operation		Execution Cost	Weight
Code length (*N* lines = Cost 1)		1	1
Control statement	if-else	1	2
	for		
	while		
	switch		
Data processing statement	stub.GetState	1	3

**Table 3:** Modules and Implemented API.

Existing Module		Implemented API	
Module Name	Description	Method	Feature
Lifecycle	Perform chaincode operations and manage admin status	GET /fabric/lifecycle/commit	Query the committed chaincode definitions
Peer	Manage CLI and peer versions	GET /fabric/peer	Get the current peer binary version
Network	Manage blockchain network status	POST /fabric/network/up	Start Fabric network with existing settings
Chaincode	Operate chaincode	POST /fabric/chaincode/query	Get endorsed result of chaincode function call and print it
Repository	Manage external modules	GET /fabric/repository/pull	Pull changes from SCM

## Data Availability

The data used to support the findings of this study are available from the authors upon reasonable request.
